# Diversity of the Microbiota of Traditional Izmir Tulum and Izmir Brined Tulum Cheeses and Selection of Potential Probiotics

**DOI:** 10.3390/foods12183482

**Published:** 2023-09-19

**Authors:** Ziba Güley, Vincenzo Fallico, Raul Cabrera-Rubio, Daniel O’Sullivan, Mariarosaria Marotta, Vincenzo Pennone, Sandra Smith, Tom Beresford

**Affiliations:** 1 Teagasc Food Research Centre, Moorepark, Fermoy, P61C996 Co. Cork, Ireland; vincenzo.fallico@gmail.com (V.F.); cabrerarubio.raul@gmail.com (R.C.-R.); osullivan_daniel_john@lilly.com (D.O.); marybios@yahoo.it (M.M.); vincenzopennone@hotmail.it (V.P.); segemba@yahoo.co.uk (S.S.); tom.beresford@teagasc.ie (T.B.); 2 Department of Food Engineering, Alanya Alaaddin Keykubat University, 07425 Antalya, Türkiye; 3 APC Microbiome Ireland, University College Cork, T12Y120 Cork, Ireland; 4 School of Food and Nutritional Sciences, University College Cork, T12K8AF Cork, Ireland

**Keywords:** high-throughput sequencing, cheese microbiota, *Lacticaseibacillus paracasei*, *Streptococcus thermophilus*, *Streptococcus infantarius*, probiotics

## Abstract

High-throughput DNA sequencing (HTS) was used to study the microbial diversity of commercial traditional Izmir Tulum (IT) and Izmir Brined Tulum (IBT) cheeses from Izmir, Türkiye. Simultaneously, cultivation-dependent methods were used to isolate, identify and characterize bacterial strains displaying probiotic potential. At the phylum level, *Firmicutes* dominated the microbiota of both cheese types comprising >98% of the population. Thirty genera were observed, with *Streptococcus* being the most abundant genus and with *Streptococcus thermophilus* and *S. infantarius* subsp. *infantarius* being the most abundant species. Genera, including *Bifidobacterium* and *Chryseobacterium*, not previously associated with IT and IBT, were detected. IT cheeses displayed higher operational taxonomic units (OTUs; Richness) and diversity index (Simpson) than IBT cheeses; however, the difference between the diversity of the microbiota of IT and IBT cheese samples was not significant. Three *Lacticaseibacillus paracasei* strains isolated from IBT cheeses exhibited probiotic characteristics, which included capacity to survive under in vitro simulated gastrointestinal conditions, resistance to bile salts and potential to adhere to HT-29 human intestinal cells. These findings demonstrate that Tulum cheeses harbor bacterial genera not previously reported in this cheese and that some strains display probiotic characteristics.

## 1. Introduction

Microorganisms play significant roles during both production and ripening of cheese and contribute to the development of flavor, aroma and texture. Type, diversity, number and population dynamics of microorganisms in cheese vary according to the quality of raw milk, whether raw or pasteurized milk is used, starter culture combination, manufacturing technology and hygienic, environmental and ripening conditions [1]. Traditional raw milk cheeses possess a more diverse microbiome and unique flavor profiles compared to industrial cheeses [2]. However, this also exposes traditional raw milk cheeses to quality and safety risks associated with the potential growth of spoilage and/or pathogenic bacteria [3]. On the other hand, the natural microbiota of these cheeses can be a valuable source of bacteria having unique features such as probiotic properties, which can promote health benefits in consumers [4,5,6,7].

**Figure 1:**
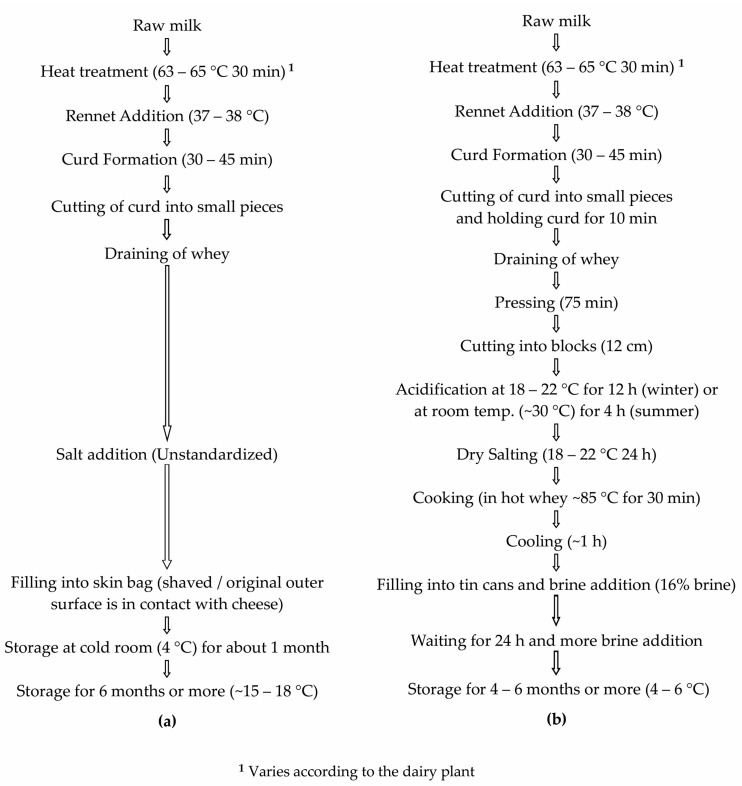
Production process of (**a**) Izmir Tulum Cheese, (**b**) Izmir Brined Tulum Cheese [9].

Tulum cheese is one of the most widely produced cheese types in Türkiye and takes its name from the goatskin casing used to package cheese during ripening. Its production is common throughout most of Türkiye. However, some differences exist in terms of production method and ripening conditions between regions. This gives rise to many varieties of Tulum cheese, one of the most popular being Izmir Tulum (IT), which is produced in the Aegean region [3,8]. IT is ripened in goatskin bags but because of the lack of sufficient supply of skin bags for industrial production, industrially produced cheese is now ripened in brine in sealed tin cans and is known as Izmir Brined Tulum (IBT) cheese. However, small-scale production of traditional IT in skin bags is still available. IT and IBT cheese also have significant differences in terms of production process. Manufactured from cow’s and/or sheep’s milk without starter culture addition, IBT cheese is acidified for 12 h; the pressed curd is cooked in hot whey (~85 °C) and ripened in 16% brine in sealed tin cans for a minimum of 4 to 6 months (Figure 1b). However, IT is dry salted, filled into skin bags after the draining of whey and does not include any cooking step (Figure 1a). 

Defining quality standards for IT and IBT cheeses is very difficult due to variations in manufacturing practices between different processors. Quality defects are reasonably common in IT and especially in IBT cheese, and consumption of the cheeses prior to completion of the required ripening period may pose risks for human health [10]. This generates the need for an accurate and detailed profiling of the microbiome of commercial examples of IT and IBT cheeses, followed by the isolation, selection and characterization of bacterial strains for inclusion in defined starter/adjunct culture blends that will enable the industrial production of cheeses with standardized quality and sensory profiles [11].

The microbiota of IT and IBT cheeses is scarcely described in the literature. Early reports used conventional culture-dependent methods where selective media were used to isolate microorganisms prior to their phenotypic identification [12,13]. However, these methods can underestimate or fail to detect low-abundance or nutritionally fastidious microorganisms [11,14]. In a more recent study, the lactic acid bacteria (LAB) microbiota diversity of IBT cheeses was determined using denaturing gradient gel electrophoresis (DGGE) in combination with real-time qPCR [15]. These molecular techniques have been shown to be valuable tools to profile microbial populations and detect previously unknown bacteria in cheese or raw milk [16,17]. The limitation of these metods is that they are laborious, have low resolution capacity and often reveal only the dominant species present in the samples [2,18].

High-throughput sequencing (HTS) of DNA has become the method of choice to investigate the microbiota and detect dominant and subdominant species of complex microbial ecosystems, including cheese [18,19,20,21,22,23,24]. No prior knowledge of the composition of the microbiome is required when applying HTS, and the fact that it is high-throughput means that many samples can be managed simultaneously in a time-efficient manner [25,26]. The application of HTS to the study of cheese ecosystems has enabled the detection of bacteria not previously associated with particular cheese types [21,27] to unveil the species causing quality defects, such as the pink discoloration defect [28], to identify populations capable of biogenic amine production [29] and to assess the effects of geography, manufacturing process, climatic conditions, seasonal variations and milk heat treatment (raw vs. pasteurized) on the cheese microbiota [1].

The objectives of this study were to:

▪ Obtain an in-depth profile of a selection of commercial IT and IBT cheeses’ microbiomes from different dairies from the Izmir province using culture independent HTS of 16S RNA genes;
▪ Simultaneously use cultivation-dependent methods to isolate, identify and characterize strains demonstrating some probiotic characteristics.

## 2. Materials and Methods

### 2.1. Cheese Samples

Commercial examples of five IT and nine IBT cheeses, collected from six different dairy plants in the Izmir Province of Türkiye, were used (for detailed information see Supplementary Material Table S1). Cheese samples (~500 g each) were vacuum-packed into sterile plastic bags, transported to the laboratory under refrigerated conditions and stored in the refrigerator (at +4 °C) until analysis.

### 2.2. Culture-Independent Profiling of IT and IBT Cheese Microbiota

#### 2.2.1. DNA Extraction from Cheeses

Five IT and five IBT cheeses were selected for the 16S rRNA gene sequence analysis. Genomic DNA was extracted using a PowerFood^®^ microbial DNA isolation kit (MoBio Laboratories Inc., Carlsbad, CA, USA), as per manufacturer’s instructions [30]. Before DNA extraction, each cheese sample was prepared and treated according to the method described by O’Sullivan et al. [29]. Quality and purity of the extracted DNA were determined using the NanoDrop 1000 spectrophotometer (Thermo Fisher Scientific, Waltham, MA, USA).

#### 2.2.2. HTS of 16S rRNA Gene Amplicons and Bioinformatic Analysis

The V3-V4 variable region of the 16S rRNA genes was amplified using the universal primers: 

5′-TCGTCGGCAGCGTCAGATGTGTATAAGAGACAGCCTACGGGNGGCWGCAG-3′ (forward) and 5′-GTCTCGTGGGCTCGGAGATGTGTATAAGAGACAGGACTACHVGGGTATCTAATCC-3′ (reverse) [31]. The resulting amplicons of 427 bp were purified and sequenced using the Ion Torrent PGM (Thermo Fisher Scientific, Waltham, MA, USA) [29]. The reads were filtered based on sequence quality (removal of low-quality nucleotides at the 3′ end) and length (removal of sequences with less than 250 bp) with PRINSEQ [32]. 

The filtered sequences were clustered at operational taxonomic unit (OTU; with 97% identity level) using UPARSE-OTU algorithm with Usearch v7.0 program [33] and removal of chimeric OTUs against GenomesOnLine database (GOLD). The taxonomic assignment of these OTUs was obtained using the ribosomal database project (RDP) [34]. The β- and α- diversity was determined using R package phyloseq [35], applying statistics by Adonis and ANOVA, respectively. *p* value greater than 0.05 was not considered significant.

### 2.3. Culture-Dependent Analysis of IT and IBT Cheeses’ Microbiota

Five grams of each of the 14 cheeses were homogenized in 45 mL of sterile 2% trisodium citrate. Ten-fold serial dilutions were prepared in Maximum Recovery Diluent (Oxoid, Basingstoke, UK), and 1 mL aliquots of appropriate dilutions were plated onto various media for the enumeration of different microbial groups and for the isolation of lactobacilli. M17 (containing lactose) (Merck, Darmstadt, Germany) agar plates incubated at 20 °C for 5 days and at 42 °C for 48 h were used for the enumeration of presumptive lactococci and thermophilic streptococci, respectively. Enterococci were counted on Kanamycin Esculin Azide Agar (KEA) (Merck, Darmstadt, Germany) incubated at 37 °C for 48 h. Overlayed BD™ LBS agar (BD, Heidelberg, Germany) was used for the enumeration and isolation of lactobacilli, incubated at 30 °C for 5 days.

### 2.4. Selection of Lactobacilli Displaying Probiotic Potential from IBT Cheese

#### 2.4.1. Isolation and Genotyping of Lactobacilli Strains

IBT cheeses from two different dairy plants and two different batches were used to mine for lactobacilli displaying probiotic potential. Colonies were randomly picked from the BD™ LBS agar and purified by streaking onto MRS agar medium twice. After microscopic examination and catalase tests, catalase negative rod-shaped isolates were genotyped at the strain level by pulsed-field gel electrophoresis (PFGE). The protocol described by Simpson et al. [36] was used to extract high molecular weight DNA and to prepare it for digestion. Digestion reactions with *AscI* restriction endonuclease were performed overnight, according to the supplier’s instructions (New England BioLabs, Hitchin, UK). The restricted DNA was loaded into the wells of a 1% PFGE-grade agarose gel and run in 0.5× Tris-borate buffer using a CHEF-DR^®^ II PFGE apparatus (Bio-Rad, Hercules, CA, USA) and the PFGE conditions stated in Güley et al. [9]. After staining with ethidium bromide, gels were visualized using an AlphaImager system (ProteinSimple, San Jose, CA, USA), and profiles were analyzed using the BioNumerics (Version 7.5) software (hierarchical clustering analysis-UPGMA) (Applied Maths, Sint-Martens-Latem, Belgium).

Isolates showing unique PFGE genotypes were identified to the species level by Sanger sequencing of the 16S rRNA gene. DNA extraction from the strains, PCR amplification of the 16S rRNA gene (primers, PCR reactions etc.), purification of PCR products and 16S rRNA gene sequencing were as described by Güley et al. [9]. Sequencing data were assembled using SeqMan Pro (DNASTAR, Madison, WI, USA) and compared to 16S rRNA gene sequences present in the National Center for Biotechnology Information (NCBI; www.ncbi.nlm.nih.gov; accessed on 5 January 2023) database using the BLASTN.

#### 2.4.2. Resistance to Simulated Gastrointestinal Tract (GIT) Conditions 

The capacity of lactobacilli isolates to transit the upper GIT was evaluated according to Pisano et al. [37], with some minor modifications. Briefly, strains were reactivated twice in 10 mL MRS broth at 37 °C to a final concentration of 10^8^–10^9^ CFU mL^−1^. Bacterial cells were recovered by centrifugation at 3000× *g* for 4 min, washed in 5 mL PBS, pH 7.4 (ThermoFisher Scientific, USA), pelletized again and finally resuspended in 10 mL of artificial gastric juice (6.2 g L^−1^ NaCl, 2.2 g L^−1^ KCl, 0.22 g L^−1^ CaCl_2_, 1.2 g L^−1^ NaHCO_3_, pH 3) containing 0.3% pepsin. After incubation at 37 °C for 90 min in a shaking incubator, 18 mL of synthetic duodenum juice (6.4 g L^−1^ NaHCO_3_, 0.239 g L^−1^ KCl, 1.28 g L^−1^ NaCl, pH 7.2), containing 0.1% pancreatin and 1.5 mL of 10% (*w*/*v*) oxgall (Sigma, Ireland), were added to the cell suspension to simulate passage into the upper small intestine. Incubation was continued at 37 °C for 90 min. One mL samples were taken immediately after (i) resuspension in simulated gastric juice, (ii) 90 min exposure to simulated gastric juice and (iii) 90 min exposure to simulated duodenum juice. Samples were serially diluted in MRD, pour-plated using MRS agar and incubated anaerobically at 37 °C for 48 h to enumerate the surviving cells. Experiments were done in triplicate, and results were expressed as the mean log CFU mL^−1^.

#### 2.4.3. Bile Salt Hydrolase Activity

Bile salt hydrolase (BSH) activity was determined according to the method described by Pisano et al. [37]. *Limosilactobacillus reuteri* NCIMB 30242 was used as positive control, whereas MRS agar plates without taurodeoxycholic acid sodium salt (TDCA)/glycodeoxycholic acid (GDCA) supplementation were used as negative controls.

#### 2.4.4. Antimicrobial Activity

The ability of the lactobacilli strains to inhibit the growth of food-related pathogens was investigated using the spot-on-lawn method as described by Bolocan et al. [38]. The pathogens used for the test (*Escherichia coli* O157:H7 P1432* and NCTC 12900‡, *Salmonella typhimurium* DPC6046* and 3784*, *Staphylococcus aureus* S17* and *Listeria monocytogenes* DPC6179*) were obtained from the Teagasc Food Research Center, Moorepark, Culture Collection* and the National Collection of Type Cultures‡ (London, UK). The presence of a distinct inhibition zone around the spots was considered as positive antagonistic effect. The inhibitory activity (IA) was calculated by subtracting the circle diameter (CD, mm) of the lactobacilli colony spreading zone from the inhibition zone diameter observed (IZD, mm) as follows: IA = (IZD − CD)/2 [39].

The inhibitory activity of the lactobacilli strains was also tested using the agar well diffusion method [40]. Cell-free supernatant (CFS) of each strain, grown in sodium acetate-free MRS broth overnight at 30 °C, was obtained by centrifugation at 3000 rpm for 4 min at 4 °C. To assess the contribution of organic acids to the activity observed, the pH of each CFS was adjusted to 6.5–7.0 with 4 mol L^−1^ NaOH and filter sterilized (0.20 μm cellulose acetate, Sigma-Aldrich, Ireland) before tested. In all assays, the nisin-A producer *Lactococcus lactis* subsp. *lactis* NZ9700 was included as a positive control. Analyses were performed in duplicate for each method.

### 2.5. In-Depth Characterization of GIT-Resistant Lactobacilli Strains Displaying Probiotic Potential

#### 2.5.1. Adhesion Properties

Lactobacilli strains showing resistance to upper GIT conditions, inhibition of pathogens and bile salts resistance were assessed for their capacity to colonize the human intestine by using the human colon adenocarcinoma cell line HT-29 as per Ross et al. [41]. Adhesion rate was calculated as the percentage of bacteria adhered to HT-29 cells compared to the initial number of bacteria added. Experiments were done in triplicate. *Lacticaseibacillus rhamnosus* GG (ATCC 53103) (LGG) was used in parallel as a positive control.

#### 2.5.2. Biogenic Amines Production

The method described by Bover-Cid et al. [42] was used to screen the strains for the potential to produce the biogenic amines tyramine and histamine.

#### 2.5.3. Antibiotic Susceptibility

The antibiotic susceptibility of the strains was determined according to the method described by Campedelli et al. [43] using VetMIC™ plates (National Veterinary Institute, Uppsala, Sweden) containing serial 2-fold dilutions of 16 antibiotics. The lowest antibiotic concentration at which no visible growth occurred was defined as the minimum inhibitory concentration (MIC) for each antibiotic. Results were interpreted based on the microbiological cut-off values established by European Food Safety Authority [44] for *Lacticaseibacillus paracasei.* Cut-off values for antibiotics not covered by EFSA were adopted from Ammor et al. [45] and Danielsen et al. [46]. When the MIC value for a specific antibiotic was higher than the corresponding microbiological cut-off value, the strain was classified as resistant [44]. Tests were performed in triplicate.

### 2.6. Statistical Analysis

Statistical analysis were carried out using the IBM SPSS^®^ (Version 27.0) software platform for Windows. One Way ANOVA/Tukey’s HSD post hoc test was used to determine statistically significant differences between the control and test strains in adhesion assay. To determine differences between the microbial counts of IT and IBT cheeses, independent samples *t* test was used. Data were analysed at the significance level of *p* < 0.05.

## 3. Results

### 3.1. HTS Shows That IT and IBT Cheeses Are a Reservoir of Streptococcus and Lactobacillaceae Species

For this study 10 commercial IT (n = 5) and IBT (n = 5) cheeses were used. Following quality filtration and length trimming of the raw data, an average of 44,403 (±8.613 SD) high-quality sequences of 16S rRNA gene was obtained for each sample. 

Phylogenetic analysis revealed that Firmicutes dominated both IT and IBT cheeses and contain very low levels of *Actinobacteria, Bacteroidetes* and *Proteobacteria*. At the genus level, 30 genera were determined. Bacteria belonging to *Streptococcus, Lacticaseibacillus, Lactobacillus*, *Lactococcus*, *Enterococcus* and *Bifidobacterium* genera were observed in all samples. Of these, *Streptococcus* was the predominant genus followed by *Lacticaseibacillus*, *Lactobacillus* and *Lactococcus*. The other genera were always detected at low levels (Table 1).

**Figure 2:**
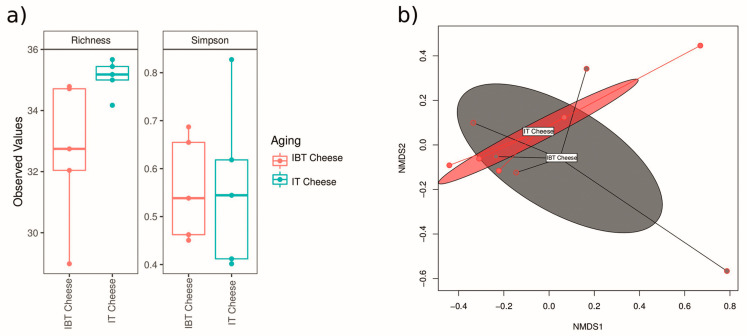
α-diversity index (**a**) and β-diversity (**b**) of the microbiota of IT and IBT cheeses.

Alpha diversity describes the diversity within an ecosystem, and it has two components: species richness and evenness [47]. IT cheeses displayed higher Operational Taxonomic Units (OTUs; Richness) and diversity index (Simpson) than IBT cheeses; however, the level of α-diversity across the group of samples was not significant in all indexes (Figure 2a). Beta diversity describes the species diversity between two ecosystems, and there are several matrixes to measure β-dversity. The Bray-Curtis dissimilarity index measures the compositional dissimilarity between the microbial communities of two groups and is based on counts on each group [48]. The β-diversity represented by non-metric multidimensional scaling (NMDS) performed using all 16S rRNA gene reads clustering all reads at 97% similarity with the Bray-Curtis distance matrix (Figure 2b). The results showed that the difference between the microbiota of the two cheese types (IT and IBT) was not significant. 

**Figure 3:**
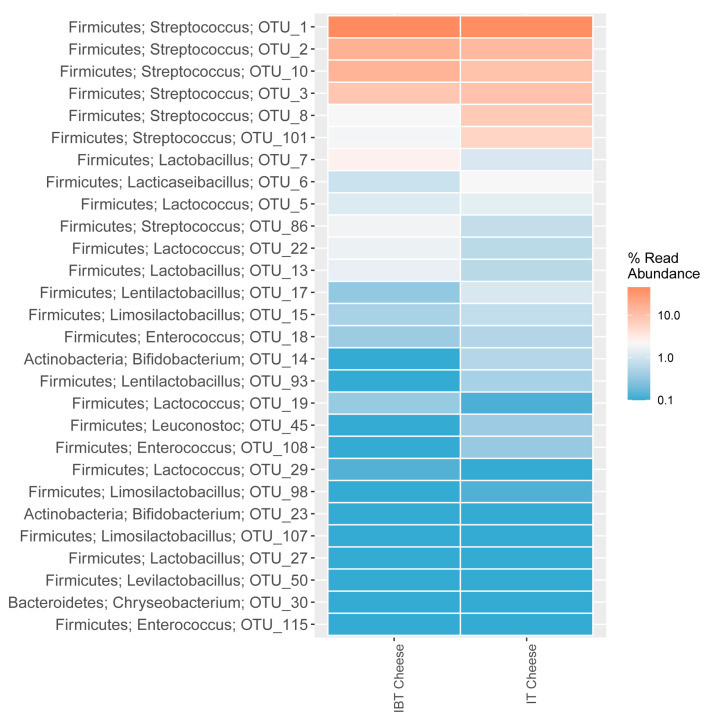
Distribution of the 28 most abundant OTUs of IT and IBT cheeses, assigned to phylum and genus level.

The abundance of *Streptococcus* was very high in both cheese types. However, differences in terms of OTU type and abundance were observed, for example OTU1 (*Streptococcus*) was highly abundant in both cheese types while OTU8 and OTU86 were only abundant in IT and IBT, respectively (Figure 3). On the other hand, although the differences were not significant statistically, there were inequalities between the populations of *Latobacillus* (OTUs 7 and 13), *Bifidobacterium* (OTU 14) and *Leuconostoc* (OTU 45) of cheese groups (Figure 3).

**Figure 4:**
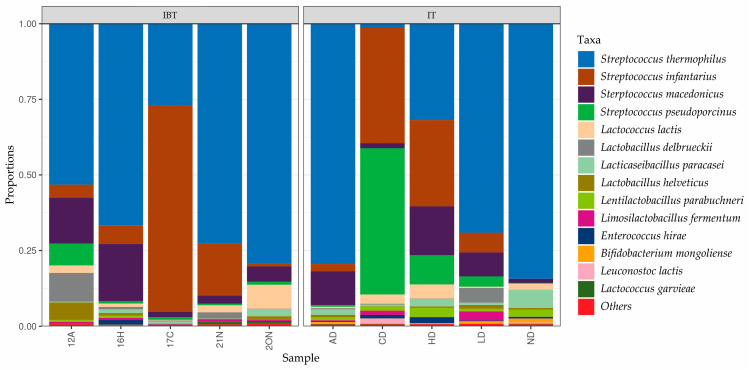
Relative abundance (%) of sequences identified at species level in IT and IBT Cheeses.

Taxonomic details up to the species level within the *Streptococcus* genus revealed that *Streptococcus thermophilus* was the most abundant OTU in most cheese samples, followed by *S. infantarius*. In some cheese samples, OTUs belonging to *S. infantarius* were higher than or equal to the *S. thermophilus*. Within the lactobacilli *Lactobacillus delbrueckii* and *Lacticaseibacillus paracasei* was the most abundant OTUs (Figure 4) observed.

### 3.2. Microbial Counts of IT and IBT Cheeses

The results of microbial counts in IT and IBT cheeses are presented in Table 2. No statistically significant difference between the bacterial counts of IT and IBT cheeses, in all agar media, was found (*p* > 0.05). These data suggest that species of the genera *Streptococcus, Lactococcus*, *Enterococcus* and lactobacilli are present in IT and IBT cheeses at similar levels and compose the dominant microbiota of these Tulum cheeses.

### 3.3. Isolation and Identification of Lactobacilli

**Figure 5:**
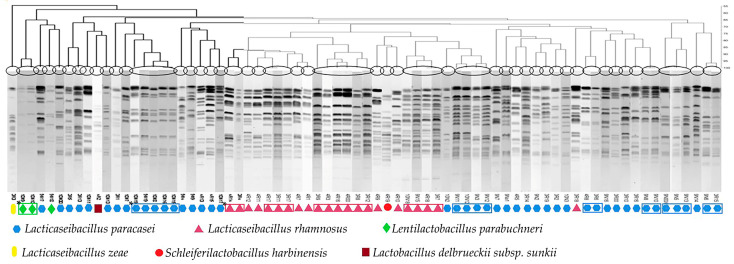
Dendrogram showing PFGE band patterns of 73 lactobacilli isolates from IBT cheeses, divided into 49 pulsotypes after *AscI* restriction. * Isolates illustrated within the boxes display similar pulsotypes.

As IBT cheeses are now the most commonly produced Tulum cheeses in the Izmir region, we focused our study on lactobacilli with probiotic characteristics in these cheeses. A total of 73 catalase-negative rod-shaped bacteria were isolated from LBS plates of IBT cheeses, purified and genotyped by PFGE. Clustering analysis of *AscI* restriction fingerprints revealed 49 unique PFGE pulsotypes (Figure 5). 16S rRNA gene sequencing of a representative of each pulsotype revealed that the isolates belonged to four genera and six different species. Specifically, the 49 PFGE pulsotypes included strains of *Lacticaseibacillus paracasei* (n = 33), *Lacticaseibacillus rhamnosus* (n = 11), *Lentilactobacillus parabuchneri* (n = 2), *Lactobacillus delbrueckii* subsp. *sunkii* (n = 1), *Schleiferilactobacillus harbinensis* (n = 1) and *Lacticaseibacillus zeae* (n = 1) (Figure 5).

### 3.4. Investigation of the Probiotic Characteristics of the Lactobacilli Isolates

The representative isolates of the 49 lactobacilli strains were investigated for a range of typical probiotic characteristics. In terms of ability to withstand the harsh GIT conditions, six *Lacticaseibacillus paracasei* strains and two *Lacticaseibacillus rhamnosus* strains showed resistance to simulated gastric and duodenum juices with survival levels matching or exceeding those of the well-known probiotic strain *Lacticaseibacillus rhamnosus* GG, which was used as control (Table 3). These strains were able to grow on MRS supplemented with TDCA, which is indicative of resistance to this bile salt. Weak growth of some strains was also observed on MRS supplemented with GDCA, whereas none of them demonstrated the ability to deconjugate bile salts, but the same was also observed for LGG.

In terms of desirable antimicrobial activities, most of the strains inhibited a range of food-related pathogens including *Staphylococcus aureus*, *Escherichia coli*, *Listeria monocytogenes* and *Salmonella*. Of the 8 GIT-resistant strains, *Lacticaseibacillus paracasei* 4R15, 9N2, 9N14, 3R2 and *Lacticaseibacillus rhamnosus* 4R11 showed inhibitory activity against all tested pathogens (Table 4). The results of both methodologies used indicated that acid production was responsible for the pathogens’ inhibition.

**Figure 6:**
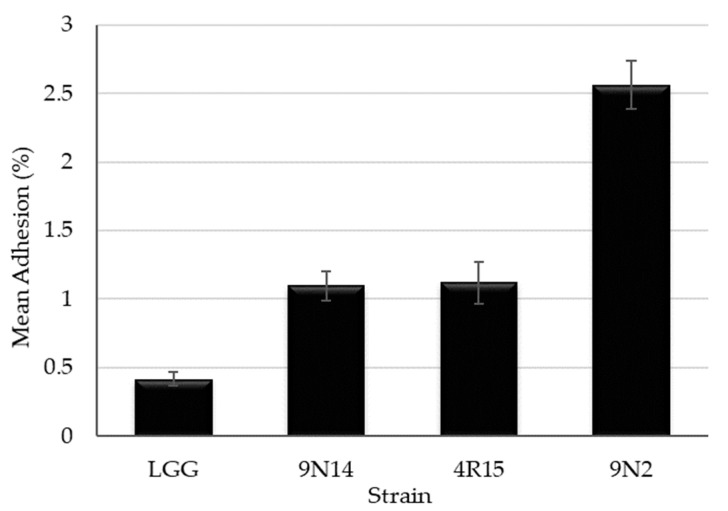
Adhesion ability to HT-29 human colon cell lines of *Lacticaseibacillus paracasei* strains with probiotic potential (mean ± standard error of three independent experiments performed in triplicate).

When tested for the ability to adhere to the intestinal epithelium cell line HT-29, three *Lacticaseibacillus paracasei* strains (4R15, 9N2 and 9N14) were found to possess adhesion properties exceeding those of the probiotic strain LGG (Figure 6). *Lacticaseibacillus paracasei* 9N2 strain was found to have the highest adherence values that were statistically significantly different (*p* < 0.05) from LGG and other *Lacticaseibacillus paracasei* strains (9N14 and 4R15).

The *Lacticaseibacillus paracasei* strains 4R15, 9N2 and 9N14 were further characterized for presence of undesirable traits, such as biogenic amine production and resistance to antibiotics. Importantly, none of these strains was found to produce the biogenic amines tyramine and histamine. In terms of antibiotic susceptibility, all three strains were resistant to vancomycin and trimethoprim, and only *Lacticaseibacillus paracasei* 9N14 was resistant to chloramphenicol. Strains were sensitive to the remaining antibiotics tested (Table 5).

## 4. Discussion

There are few reports concerning the whole microbiota of IT and IBT cheeses. Species belonging to *Enterococcus*, *Lactobacillus*, *Leuconostoc* and *Lactococcus* genera were isolated and identified during these studies using conventional culture dependent methods [12,13]. *E. durans*, *E. faecalis*, *E. faecium* and *L. casei* (now *Lacticaseibacillus casei*) were found as dominant species. Other *Lactobacillus* spp., *Lactococcus lactis* subsp. *lactis*, *L. lactis* subsp. *cremoris*, and *Leuconostoc* spp. were also identifed in both IT and IBT cheeses [12,13]. Apart from these, more recently, *Streptococcus thermophilus*, *Lactococcus lactis* subsp. *lactis*, *Lactobacillus gallinarum*, *Streptococcus equinus*, *Streptococcus infantarius* subsp. *infantarius*, *E. faecalis*, *E. faecium* and *Lactococcus garvieae* were found as dominant species using the DGGE method [15].

In the present study, microbiota of both IT and IBT cheeses were investigated using the HTS approach, for the first time, in order to provide insights into the microbiota of these cheeses and to examine if differences between IT and IBT cheese could be identifed (Figure 1). Taxonomic classification of the DNA sequence data identifed mainly four phyla: Firmicutes, Actinobacteria, Bacteroidetes and Proteobacteria in IT and IBT cheeses, which is in agreement with the findings of other works on cheeses [18,20,27]. Similarly, as with the previous reports, at phylum level Firmicutes were the primary microbiota of both cheese types. Besides known genera, the method effectively revealed the presence of a number of other genera not previously associated with IT and IBT cheeses. Different from the former findings on IT and IBT cheeses, *Streptococcus* was found as the dominant genus in all cheese samples. OTUs belonging to *Lacticaseibacillus*, *Lactobacillus*, *Enterococcus* and *Lactococcus* genera were also detected in all samples but as relatively low proportions of the overall bacterial population. Besides the identification method used, several factors could be responsible for this difference. It is well established that animal source of milk, pasteurization, raw milk microbiota, production environment and production process parameters, salt content, etc. influence microbial populations present in the resultant cheese [19,27]. Any change in these parameters alter the equilibrium of cheese microbiota. From the time when the original studies were undertaken on these cheeses [8,49] to the present investigation, many changes have occurred to milk production technology, handling and transportation systems, hygiene practices, equipments and even IT and IBT cheese production processes. For example, pasteurization/thermization of milk and immersion of the pressed curd blocks into hot whey (in the region of 85 °C) for 30 min, the so called cooking step, were added to the production process of IBT cheese (Personal communication). Quigley et al. [27] observed a significant difference in the levels of *Lactococcus* and *Lactobacillus* between unpasteurized and pasteurized milk cheeses. Also, by comparing the bacterial genera present in artisanal cheeses manufactured from unpasteurized milk relative to those made from pasteurized milk, they detected some genera that were present in raw milk cheeses only, while some others were unique for pasteurized milk cheeses [27].

Taxonomic details up to the species level within the *Streptococcus* genus revealed that *Streptococcus thermophilus* and *Streptococcus infantarius* (*S. infantarius* subsp. *infantarius*) were the most abundant OTUs (Figure 4). This is consistent with the findings of recent studies on IBT cheese. Karabey et al. [15] detected *Streptococcus thermophilus* and *Streptococcus infantarius* subsp. *infantarius* amongst the dominant species of IBT cheeses. In a recent study conducted by our research team *S. infantarius* subsp. *infantarius* was determined as the species primarily responsible for acid production in IBT cheese [9]. Within the *Lacticaseibacillus* genus, *Lacticaseibacillus paracasei* were the most abundant OTU, which is consistent with the results of culture-dependent identification.

The culture-independent approach used in this study identified other genera for the first time in mature IT and IBT cheeses. Among these, *Bifidobacteria* and *Chryseobacterium* were detected in all samples while others were observed in some of them. 

Members of the *Chryseobacterium* genus are gram negative, non-spore-forming, proteolytic, psychrotrophic bacteria that are widely distributed in a variety of environments, such as fresh water, sewage, soil and foods [50]. *Chryseobacterium* spp. have been frequently described in raw milk and in cheese among subdominant genera [18,27,51]. *Chrysobacterium* have the capacity to produce hydrolytic thermostable enzymes that lead to formation of undesirable aroma and flavor compounds during milk storage and cheese ripening, and thus, there is concern that they have the potential to cause spoilage defects in dairy products [52]. Further investigations are required to determine the source of these bacteria in IT and IBT cheeses, the species present in the cheese and their contribution to the maturation of the cheeses in order to understand whether they impact on key quality attributes of these cheeses.

The presence of bifidobacteria and *Bifidobacterium mongoliense* in some traditional and artisanal raw milk cheeses was also reported previously [19,27,53]. Bifidobacteria in cheese probably originates from raw milk contaminated with animal feces and may contribute to some organoleptic and technological characteristics [53]. The growth characteristics and nutrient requirements of bifidobacteria are different from most LAB. They have low proteolytic activity and usually require an anaerobic environment to survive. Their growth and long-term survival in cheese is supported by LAB metabolism, which alters cheese pH, limits oxygen levels and provides growth promoters [54]. It has been shown that *S. thermophilus* strains with high oxygen consumption ability enhance the viability of bifidobacteria [54]. In the present study, the presence of *Bifidobacteria* in all IT and IBT cheese samples may be linked to the high levels of *Streptococcus thermophilus* found in the cheese. *Bifidobacterium* ssp. are widely used as probiotic microorganisms. *Bifidobacterium mongoliense* strains showing in vitro resistance to gastric and pancreatic juices, and bile salts, and having ability to digest milk oligosaccharides and produce antivirulent metabolites, have been reported [53,55]. Therefore, efforts should be made in the future to recover and investigate the probiotic potential of bifidobacteria from IBT and IT cheeses.

Culture dependent microbial counts of cheeses exhibited different profile than metagenomic analysis. No statistical differences were observed between the bacterial counts of both IT and IBT across the four growth conditions tested implying that *Streptococcus*, *Lactococcus*, *Enteroccocus* and lactobacilli are present at similar levels and constitute the dominant LAB microbiota of IT and IBT cheeses. However, HTS data indicated that the microbiome of all cheeses were dominated by *Streptococcus*. It is well known that M17 and LBS agar (also known as Rogosa Agar) media are not very selective, and species of enterococci, lactobacilli, streptococci and lactococci are able to grow on both of these media [56,57]. KEA agar is used as a selective medium for the enumeration of enterococci. Growth of some *Lactobacillus* and *Pediococcus* species on KEA agar, with colony structure similar to enterococci, has been observed [58]. Therefore, the lack of selectivity could partly explain the broadly similar populations of bacteria observed under each of the four growth conditions tested. On the other hand, HTS analysis measures samples’ total DNA, which in the case of ripened cheeses certainly includes DNA originating from live, injured, dead and possibly the so-called viable-but-not-culturable cells [59]. Therefore, as we previousely demonstrated, *Streptococcus* is primarialy responsible for acid production during cheese manufacture [9] and thus present as the dominant component of the microbiome during cheese manufacture. However, by the end of ripening it is possible that many of these are no longer viable while their DNA would still be present in the cheese and being detected by HTS. Even so, the abundance of the genus *Streptococcus* in IT and IBT cheeses microbiota is obvious.

Although it has been reported that the use of different packaging materials (skinbag versus plastic, and wooden box) for Tulum cheeses affected the microbial composition of the cheeses [60], in present study no significant difference was determined between the microbiota of IT and IBT cheeses.

Traditional fermented foods, especially cheeses, can be a good source of microorganisms possessing probiotic properties [4,5,6,7]. In this respect, mesophilic lactobacilli are particularly sought after as they constitute a significant fraction of Non-Starter LAB microbiota of ripened cheeses and can find wide applications as starters, adjunct cultures and probiotics [61,62]. Therefore, when seeking to determine if bacteria from Tulum cheese may express characteristics associated with probiotics, we focused our study on mesophilic lactobacilli. 

In this study, strains of six different species belonging to four genera from the *Lactobacillaceae* family were isolated from IBT cheeses, with *Lacticaseibacillus paracasei* being the most commonly encountered followed by *Lacticaseibacillus rhamnosus*. Selected strains of *Lacticaseibacillus casei, Lacticaseibacillus paracasei* and *Lacticaseibacillus rhamnosus* have applications as probiotics and are claimed to have beneficial effects on human health [63]. 

In this study, the overall lactobacilli population of IBT cheese were investigated for their probiotic characteristics for the first time. Tolerance to GIT conditions and ability to adhere to intestinal cells are mandatory features of bacteria displaying probiotic potential [4,64]. Amongst 49 tested strains, six *Lacticaseibacillus paracasei* and two *Lacticaseibacillus rhamnosus* showed ability to remain viable following simulated GIT conditions, with survival levels matching or exceeding those of the well known probiotic strain LGG. The strains *Lacticaseibacillus paracasei* 4R15, 9N2 and 9N14, and *Lacticaseibacillus rhamnosus* 4R11 exhibited the best survival rates with less than 2.5 log reduction. Good survival ability of several *Lacticaseibacillus paracasei* [37], *Lacticaseibacillus casei* and *Lacticaseibacillus rhamnosus* strains [5] have been reported by other researchers. 

Another important function of a probiotic bacteria is to protect the host gastrointestinal tract from pathogen infection through the production of inhibitory compounds such as organic acids (e.g., lactic acid, acetic acid), hydrogen peroxide, and bacteriocins [65]. Here, the GIT-resistant strains of *Lacticaseibacillus paracasei* (4R15, 9N2, 9N14, 3R2) and *Lacticaseibacillus rhamnosus* (4R11) showed ability to inhibit all tested pathogens, and the inhibition was due to organic acids. In agreement with our findings, the antagonistic effects of *Lacticaseibacillus paracasei* and *Lacticaseibacillus rhamnosus* strains against pathogens have been associated with production of organic acids [37].

Although its contribution is not fully described, BSH activity is thought to be a necessary feature for probiotic strains to withstand the toxicity of conjugated bile salts in the duodenum and survive in the highly competitive environment of the human intestinal tract. However, some reports suggest that resistance to bile salts in lactobacilli is not always related to hydrolase activity [66]. Lactobacilli strains tested in this study were able to grow in the presence of conjugated bile salts but did not hydrolyze GDCA and TDCA. Similar characteristics were observed in lactobacilli strains isolated from fermented foods [37,66].

Adhesion to and colonization to the gastrointestinal tract of the host is another important trait of probiotic bacteria. Therefore, the ability of the strains to adhere to the human intestinal cell lines is an important criterion while evaluating the probiotic potential of novel strains [39,67]. Adhesion ability is a strain and matrix specific trait [64] with levels of adhesion ranging from 3 to 20% for various *Lacticaseibacillus casei* and *Lacticaseibacillus paracasei* strains being reported [37,68]. In our study, three *Lacticaseibacillus paracasei* strains were able to adhere to the human intestinal cell line HT-29 at levels varying from 1% to 2.6% and higher than that observed for the reference strain LGG. Similar to our findings, low adhesion levels of LGG to HT-29 cells were reported by other authors [69]. 

As they may contribute to the transfer of antibiotic resistance genes, all bacteria intended for use as probiotics or starter cultures must be assessed for their sensitivity/resistance to antibiotics of human and veterinary importance and must comply with the guidelines set out by the EFSA [44]. In the present study, all tested strains were resistant to vancomycin and trimethoprim. Vancomycin resistance is well characterized in lactobacilli and has also been reported for *Lacticaseibacillus paracasei* strains [37,39]. It is attributed to the synthesis of peptidoglycan precursors terminating with D-alanyl-D-lactate conferring vancomycin resistance. Such resistance is intrinsic, chromosomally encoded and not transferable [70]. Lactobacilli, including the *Lacticaseibacillus* species, have also been reported as being intrinsically resistant to trimethoprim [43]. In LAB, trimethoprim resistance is associated with the absence of the folic acid synthesis pathway, which is the target of this antibiotic, and it has been described as an intrinsic feature [71]. Intrinsic and acquired resistance by mutation are assumed to have a low potential of horizontal transfer [44]. Lactobacilli are generally known as susceptible to chloramphenicol; however, in recent years transferable chloramphenicol resistant genes as well as phenotypic resistance have been observed in lactobacilli [43,45]. *Lacticaseibacillus paracasei* 9N14 strain showed resistance to chloramphenicol. The strains tested in our study, except 9N14, do not seem to represent a source for transferable resistance genes since they were phenotypically susceptible to the remaining 14 antibiotics. But the absence of acquired or transferable resistance factors must be proven genotypically in these strains, especially in the strain 9N14, before used as probiotics.

Biogenic amines occur in foods as a result of amino acid decarboxylation by decarboxylase positive microorganisms and can cause toxicological effects (e.g., hypertension, headaches, palpitations and vomiting) in humans. Cheese can contain potentially harmful levels of biogenic amines, especially histamine and tyramine [72]. The EFSA regards histamine and tyramine as the most important biogenic amines from a toxicological standpoint [73]. Lactobacilli species are amongst the major biogenic amines producers in cheese, with *Lacticaseibacillus paracasei* strains being reported to produce tyramine [74]. It is important to note that the probiotic candidates in our study do not produce biogenic amines. This is a desirable trait for food grade microorganisms.

These data, taken together, demonstrate that strains of lactobacilli from Tulum cheese encode a range of characteristics required of a probiotic. They also survive in the cheese during ripening and are among the dominant strains present. These all support the view that these strains could be applied during cheese manufacture with a view to enhancing the health-promoting properties of cheese. However, the in vivo potential of these strains as probiotics would first need to be confirmed.

## 5. Conclusions

The application of HTS of DNA gave detailed new information about the microbiota of commercial examples of IT and IBT cheeses. This approach:

- Highlighted the dominance of the genus *Streptococcus* and, within the genus, the abundance of the species *S. thermophilus* and *S. infantarius* subsp. *infantarius*.
- It unveiled the presence of genera, including *Bifidobacteria* and *Chryseobacterium,* that have not been reported in these cheese types before.

Results from the culture-dependent approach confirmed *Streptococcus* as a key microbial population in IT and IBT cheeses but also demonstrated that *Lactococcus, Enterococcus* and lactobacilli are present in large populations.

This information will provide the base for further comprehensive studies to solve the quality problems and to create appropriate starter/adjunct cultures for these cheeses, in order to produce a Tulum cheese of standardized quality. 

The investigations also gave valuable information about the *Lactobacillaceae* microbiota of the IBT cheeses. Evaluation of their in vitro probiotic properties displayed the presence of a potentially highly beneficial microbiota in these traditional cheeses. Three *Lacticaseibacillus paracasei* strains exhibited favorable in vitro probiotic properties including: Potential to survive passage through the GIT, inhibition of selected pathogens, adhesion ability to human colon cells, antibiotic sensitivity, absence of biogenic amine production.

Therefore these strains could be candidates for inclusion as starter/adjunct cultures in the manufacture of Tulum cheeses or of probiotic-containing fermented foods. This is the first study that the overall lactobacilli population from IBT cheese were investigated for their probiotic characteristics. Additional studies are required to confirm their in vivo probiotic properties and technological attributes.

## Figures and Tables

**Table 1:** Relative abundance of OTUs assigned to the phylum and genus level in Izmir Tulum (IT) and Izmir Brined Tulum (IBT) cheeses.

	% of Reads									
Phyla and Genera	IT Cheeses					IBT Cheeses				
	AD	CD	HD	LD	ND	12A	16H	17C	21N	2ON
Phyla										
*Firmicutes*	99.01	99.42	99.50	99.00	98.22	99.77	99.65	99.87	99.43	99.83
*Actinobacteria*	0.76	0.08	0.17	0.82	1.58	0.07	0.22	0.03	0.24	0.06
*Bacteroidetes*	0.08	0.33	0.06	0.17	0.04	0.13	0.06	0.06	0.18	0.03
*Proteobacteria*	0.15	0.13	0.17	0.01	0.16	0.04	0.03	0.01	0.13	0.03
*Tenericutes*	0	0	0.05	0	0	0	0	0	0	0.03
Others	0	0.02	0.03	0	0	0	0.04	0.03	0.01	0.02
Genera										
*Streptococcus*	93.65	88.28	86.13	87.15	87.39	80.00	92.40	97.53	92.65	86.65
*Lacticaseibacillus*	1.77	0.54	2.77	0.68	4.81	0.21	1.24	0.71	0.07	2.23
*Lactobacillus*	1.45	0.44	0.60	6.04	0.64	15.21	1.78	0.38	2.17	0.97
*Lactococcus*	0.65	3.30	4.94	0.50	2.30	3.14	1.22	0.71	3.57	8.37
*Lentilactobacillus*	0.75	2.03	2.46	0.73	2.03	0.53	0.65	0.14	0.01	0.33
*Limosilactobacillus*	0.33	1.59	0.09	2.97	0.04	0.51	0.77	0.20	0.89	0.55
*Enterococcus*	0.31	1.47	2.08	0.38	0.72	0.16	1.53	0.05	0.01	0.31
*Bifidobacterium*	0.75	0.06	0.15	0.81	1.56	0.05	0.19	0.02	0.21	0.04
*Leuconostoc*	0.03	1.71	0.12	0.01	0.04	0.03	0.01	0.02	0.01	0.01
*Chryseobacterium*	0.07	0.28	0.05	0.14	0.03	0.12	0.05	0.05	0.16	0.03
*Schleiferilactobacillus*	0.04	0.01	0.02	0.02	0.05	0.02	0.05	0.002	0.006	0.01
*Levilactobacillus*	0.02	0	0.10	0	0.01	0	0	0	0	0.38
*Latilactobacillus*	0.004	0.02	0.004	0.002	0.02	0	0	0	0.01	0.002
*Companilactobacillus*	0	0.003	0	0.31	0.01	0	0	0	0	0
*Secundilactobacillus*	0	0.003	0	0.04	0	0	0	0	0.002	0
*Loigolactobacillus*	0	0	0	0.02	0.13	0	0	0	0	0.002
*Pediococcus*	0	0.01	0	0.19	0.002	0	0.01	0.11	0	0.002
*Weissella*	0.004	0	0.007	0.01	0.008	0	0.005	0	0	0.004
*Macrococcus*	0	0.02	0.18	0.002	0.002	0.02	0.005	0	0	0.02
*Acinetobacter*	0.01	0.05	0	0	0.01	0.02	0.009	0.007	0.03	0.006
*Escherichia-Shigella*	0.06	0.03	0.01	0	0	0.003	0.005	0	0.05	0.002
*Sphingobacterium*	0.004	0.02	0.007	0.02	0.008	0.01	0.01	0.01	0.02	0
*Bacillus*	0	0	0	0	0	0	0	0.002	0.01	0
*Microbacterium*	0.004	0	0.01	0.002	0.004	0.005	0.03	0.01	0.007	0.002
*Schaalia*	0	0.006	0	0.002	0	0.003	0	0	0.002	0
*Elizabethkingia*	0	0.03	0	0.002	0	0	0	0	0	0
*Empedobacter*	0	0	0	0.004	0.003	0	0	0	0.002	0
*Enhydrobacter*	0	0	0	0.002	0	0.01	0	0.002	0.002	0
*Rothia*	0.006	0.02	0.01	0.004	0.005	0.006	0	0.002	0.01	0.02
*Mycoplasmopsis*	0	0	0.05	0	0	0	0	0	0	0.03
Others	0.09	0.07	0.20	0.01	0.16	0.01	0.05	0.04	0.07	0.03

**Table 2:** Microbial counts (log_10_ CFU g ^−1^) in Izmir Tulum (IT) and Izmir Brined Tulum (IBT) cheeses.

		Counts (log_10_ CFU g ^−1^)			
Cheese Samples		*Lactococcus*	*Streptococcus*	*Lactobacilli*	*Enterococcus*
IT	AD	7.30	7.39	7.05	6.75
	CD	7.51	7.61	5.00	6.63
	HD	7.93	8.06	6.95	7.59
	LD	7.21	7.26	7.35	6.98
	ND	7.24	7.20	5.07	6.41
IBT	12A	8.70	7.78	7.88	6.95
	16H	8.09	8.18	7.03	7.91
	17C	6.33	6.94	6.05	6.00
	21N	7.00	7.08	6.11	4.85
	20 N	7.25	7.44	7.04	7.05
	9N	8.44	8.60	7.96	7.63
	10N	8.07	8.55	8.09	7.49
	3R	7.88	7.85	7.26	7.54
	4R	7.57	7.79	7.70	7.48

**Table 3:** Survival of selected *Lactobacillaceae* strains and *Lacticaseibacillus rhamnosus* GG (control) following exposure to simulated GIT conditions (mean ± standard error of three independent experiments).

Strains	Initial Mean Counts (log_10_ CFU mL^−1^)	Counts after 90 min in Simulated Gastric Juice at pH 3.0 (log_10_ CFU mL^−1^)	Counts after 180 min in Simulated Gastric and Duodenum Juices at pH 7.2 (log_10_ CFU mL^−1^)	Log Reduction
*L. rhamnosus* GG	8.39 ± 0.03	8.99 ± 0.01	4.10 ± 0.10	4.29
*L. paracasei* 3R00	8.26 ± 0.12	8.53 ± 0.11	5.11 ± 0.42	3.15
*L. paracasei* 3R2	8.31 ± 0.06	8.31 ± 0.64	4.91 ± 0.93	3.40
*L. rhamnosus* 4R11	8.31 ± 0.08	9.13 ± 0.00	6.21 ± 0.01	2.10
*L. rhamnosus* 4R12	8.50 ± 0.12	9.07 ± 0.04	5.60 ± 0.27	2.90
*L. paracasei* 4R15	8.47 ± 0.49	8.91 ± 1.18	6.02 ± 0.56	2.45
*L. paracasei* 9N2	8.21 ± 0.13	8.56 ± 0.10	6.46 ± 0.10	1.75
*L. paracasei* 9N4	7.39 ± 0.58	6.45 ± 0.37	4.62 ± 1.15	2.77
*L. paracasei* 9N14	8.67 ± 0.03	9.27 ± 0.06	7.28 ± 0.18	1.39

**Table 4:** Inhibitory activity of potential probiotic strains of *Lacticaseibacillus paracasei* and *Lacticaseibacillus rhamnosus* against selected pathogens ^1^ (mean of two independent experiments).

	Zone of Inhibition (mm)							
		*L. paracasei*					*L. rhamnosus*	
Target Pathogens	9N14	4R15	9N2	9N4	3R00	3R2	4R11	4R12
*E. coli* O157:H7 P1432	3	6	5	4	0	5	3	4
*E. coli* O157:H7 12900	2	3	4	0	0	4	2	0
*S. aureus* S17	2	5	4	2	0	4	4	4
*L. monocytogenes* DPC 6179	6	7	5	4	0	5	4	4
*S. typhimurium* 3784	2	5	5	3	0	5	2	2
*S. typhimurium* DPC 6046	2	3	3	2	0	3	2	2

^1^ Results obtained with the spot-on-lawn method.

**Table 5:** VetMIC™ values (MIC as µg mL^−1^) * obtained for three *Lacticaseibacillus paracasei* strains isolated from IBT Cheeses and their comparison with the microbial cut-off values (µg mL^−1^) established by EFSA ^a^ [44], and reported by Ammor et al. ^b^ [45] and Danielsen et al. ^c^ [46] for *Lacticaseibacillus casei/paracasei*.

	Antibiotics															
Strains	Ampicillin	Vancomycin	Gentamicin	Kanamycin	Streptomycin	Erythromycin	Clindamycin	Tetracycline	Chloramphenicol	Trimethoprim	Penicillin	Quinupristin-Dalfopristin	Linezolid	Ciprofloxacin	Rifampicin	Neomycin
*L.casei/L. paracasei*	4 ^a^	n.r. ^1 a^	32 ^a^	64 ^a^	64 ^a^	1 ^a^	1 ^a^	4 ^a^	4 ^a^	8 ^b^	4 ^c^	4 ^b^	4 ^b^	>32 ^c^	2 ^c^	16 ^b^
9N2	2	>128 **	4	64	32	0.12	0.5	1	4	**>64**	1	2	2	4	0.5	4
9N14	2	**>128**	4	64	32	0.25	0.25	1	**16**	**>64**	1	2	4	4	1	8
4R15	2	**>128**	16	64	32	0.25	0.5	1	4	**>64**	1	2	2	2	0.5	8

^1^ n.r. not required (indicated by EFSA), * Value obtained from at least two replicates of the VetMIC experiments, done in triplicate. ** MIC (µg mL^−1^) values in bold indicate presence of antibiotic resistance.

## Data Availability

Data is contained within the article or supplementary material. Extra data will be provided on request.

## Supplementary Materials

The following supporting information can be downloaded at: https://www.mdpi.com/article/10.3390/foods12183482/s1, Table S1: Cheese samples used in the study.
