# Supplementary material for: Diversity of the Microbiota of Traditional Izmir Tulum and Izmir Brined Tulum Cheeses and Selection of Potential Probiotics
Source: Foods. 2023 Sep 19;12(18):3482. doi: 10.3390/foods12183482 (PMC10528788; doi:10.3390/foods12183482)
Supplement: Supplementary file 1 [file foods-12-03482-s001.zip › foods-2585236-supplementary.pdf]

Table S1. Cheese samples used in the study

<table>
<tr><th rowspan="2">Dairy Code</th><th rowspan="2">Sample Code</th><th rowspan="2">Cheese Type</th><th rowspan="2">Aging</th><th rowspan="2">Age (months)</th><th colspan="3">Analysis Applied to Cheese Samples</th></tr>
<tr><th>Culture-independent analysis</th><th>Culture-dependent analysis</th><th>Mining for lactobacilli strains with probiotic characteristics</th></tr>
<tr><td>A</td><td>AD</td><td>IT*</td><td>Skin bag</td><td>14</td><td rowspan="10">YES</td><td rowspan="14">YES</td><td rowspan="10">NO</td></tr>
<tr><td>C</td><td>CD</td><td>IT</td><td>Skin bag</td><td>10</td></tr>
<tr><td>H</td><td>HD</td><td>IT</td><td>Skin bag</td><td>22</td></tr>
<tr><td>L</td><td>LD</td><td>IT</td><td>Skin bag</td><td>14</td></tr>
<tr><td>N</td><td>ND</td><td>IT</td><td>Skin bag</td><td>14</td></tr>
<tr><td>A</td><td>12A</td><td>IBT**</td><td>Brine/Can</td><td>8</td></tr>
<tr><td>H</td><td>16H</td><td>IBT</td><td>Brine/Can</td><td>10</td></tr>
<tr><td>C</td><td>17C</td><td>IBT</td><td>Brine/Can</td><td>10</td></tr>
<tr><td>N</td><td>20N</td><td>IBT</td><td>Brine/Can</td><td>8-9</td></tr>
<tr><td>N</td><td>21N</td><td>IBT</td><td>Brine/Can</td><td>8-9</td></tr>
<tr><td>N</td><td>9N</td><td>IBT</td><td>Brine/Can</td><td>8</td><td rowspan="4">NO</td><td rowspan="4">YES</td></tr>
<tr><td>N</td><td>10N</td><td>IBT</td><td>Brine/Can</td><td>8</td></tr>
<tr><td>R</td><td>3R</td><td>IBT</td><td>Brine/Can</td><td>8</td></tr>
<tr><td>R</td><td>4R</td><td>IBT</td><td>Brine/Can</td><td>8</td></tr>
</table>

* Izmir Tulum Cheese ** Izmir Brined Tulum Cheese
